# Hemojuvelin and bone morphogenetic protein (BMP) signaling in iron homeostasis

**DOI:** 10.3389/fphar.2014.00104

**Published:** 2014-05-13

**Authors:** Amanda B. Core, Susanna Canali, Jodie L. Babitt

**Affiliations:** Division of Nephrology, Program in Membrane Biology, Center for Systems Biology, Massachusetts General Hospital, Harvard Medical School, Program in Anemia Signaling ResearchBoston, MA, USA

**Keywords:** hemojuvelin, bone morphogenetic protein, hepcidin, iron, hemochromatosis, repulsive guidance molecule

## Abstract

Mutations in hemojuvelin (HJV) are the most common cause of the juvenile-onset form of the iron overload disorder hereditary hemochromatosis. The discovery that HJV functions as a co-receptor for the bone morphogenetic protein (BMP) family of signaling molecules helped to identify this signaling pathway as a central regulator of the key iron hormone hepcidin in the control of systemic iron homeostasis. This review highlights recent work uncovering the mechanism of action of HJV and the BMP-SMAD signaling pathway in regulating hepcidin expression in the liver, as well as additional studies investigating possible extra-hepatic functions of HJV. This review also explores the interaction between HJV, the BMP-SMAD signaling pathway and other regulators of hepcidin expression in systemic iron balance.

## Juvenile hemochromatosis is caused by mutations in the genes encoding hepcidin or hemojuvelin

Juvenile Hemochromatosis (JH) is an autosomal recessive disorder caused by a failure to prevent excess iron entry into the bloodstream, and characterized by progressive tissue iron overload (Pietrangelo, 2010). Although iron's redox properties are critical for its role in many fundamental biological processes from cellular respiration to oxygen transport, iron excess can lead to toxic free radical generation. If left untreated, JH patients develop multiorgan dysfunction as a consequence of iron overload, including cirrhosis, cardiomyopathy, diabetes mellitus, and hypogonadotrophic hypogonadism, before the age of 30 (Pietrangelo, 2010).

The identification of hepcidin as a master regulator of systemic iron balance was a major advance in understanding the pathophysiology of JH (Ganz, 2013). A defensin-like peptide produced predominantly by hepatocytes, hepcidin controls iron entry into the bloodstream from dietary sources, recycled red blood cells, and body storage sites by inducing degradation of the iron exporter ferroportin (Ganz, 2013). Hepcidin expression is stimulated by iron and inflammation to limit iron availability, while hepcidin is inhibited by iron deficiency, anemia, and hypoxia to increase iron availability for erythropoiesis (Babitt and Lin, 2010; Ganz, 2013). Hepcidin deficiency is the common pathogenic mechanism underlying both adult and juvenile-onset hemochromatosis and contributes to the pathogenesis of iron loading anemias such as thalassemia, while its overproduction causes anemia of inflammation and iron refractory iron deficiency anemia (IRIDA) (Ganz, 2013). JH is caused by mutations in the gene encoding hepcidin itself (*HAMP*) or, more commonly, hemojuvelin (*HJV*, also known as *HFE2* or *RGMC*) (Roetto et al., 2003; Papanikolaou et al., 2004).

*HJV* encodes a glycophosphatidylinositol (GPI)-linked membrane protein that is a member of the repulsive guidance molecule (RGM) family (Monnier et al., 2002; Samad et al., 2004). Currently, there are 43 identified *HJV* mutations that cause JH, with G320V being the most frequent (Table 1). HJV is expressed in the liver, and JH patients with *HJV* mutations and *Hjv* knockout mice exhibit significantly reduced hepatic hepcidin expression, thereby implicating HJV in the regulation of hepcidin synthesis (Papanikolaou et al., 2004; Huang et al., 2005; Niederkofler et al., 2005).

**Table 1:** **Mutations of the *HJV* gene linked to JH**.

**Residue mutation**	**Exon**	**Type of mutation**	**Nucleotide change**	**Family origin**	**References**
Q6H	2	Missense	18G > C	Asian	Huang et al., 2004
L27fsX51	2	Frame shift	81delG	English/Irish	Wallace et al., 2007
R54X	3	Nonsense	160A > T	African American	Murugan et al., 2008
G66X	3	Nonsense	196G > T	Romanian	Jánosi et al., 2005
V74fsX113	3	Frame shift	220delG	English	Lanzara et al., 2004
C80R	3	Missense	238T > C	Caucasian	Lee et al., 2004
S85P	3	Missense	253T > C	Italian	Lanzara et al., 2004
G99R	3	Missense	295G > A	Albanian	Lanzara et al., 2004
G99V	3	Missense	296G > T	Multiple	Papanikolaou et al., 2004; Silvestri et al., 2007
L101P	3	Missense	302T > C	Albanian	Lanzara et al., 2004; Lee et al., 2004
G116X	3	Nonsense			Santos et al., 2012
C119F	3	Missense	G356 > T	German	Gehrke et al., 2005; Silvestri et al., 2007
R131fsX245	3	Frame shift	391-403del	Italian	Lanzara et al., 2004
D149fsX245	3	Frame shift	445delG	Italian	Lanzara et al., 2004
L165X	3	Nonsense	494T > A		van Dijk et al., 2007
A168D	3	Missense	503C > A	Australian /English	Lanzara et al., 2004
F170S	3	Missense	509T > C	Italian	De Gobbi et al., 2002; Lanzara et al., 2004; Silvestri et al., 2007
D172E	3	Missense	516C > G	Italian	Lanzara et al., 2004
R176C	3	Missense	526C > T	European	Aguilar-Martinez et al., 2007; Ka et al., 2007
W191C	3	Missense	573G > T	Italian	De Gobbi et al., 2002; Lanzara et al., 2004; Silvestri et al., 2007
N196K	3	Missense	588T > G		Santos et al., 2012
S205R	3	Missense	615C > G	Italian	Lanzara et al., 2004
I222N	4	Missense	665T > A	Canadian	Papanikolaou et al., 2004
K234X	4	Nonsense	700-703AAG del	European	Santos et al., 2012
D249H	4	Missense	745G > C	Asian	Santos et al., 2012
G250V	4	Missense	749G > T	Italian	Lanzara et al., 2004
N269fsX311	4	Frame shift	806 > 807insA	English	Lanzara et al., 2004
I281T	4	Missense	842T > C	Multiple	Huang et al., 2004; Papanikolaou et al., 2004
C282Y	4	Missense		Caucasian	Le Gac et al., 2004
R288W	4	Missense	863C > T	French	Lanzara et al., 2004
R288Y	4	Missense	862C > T		Wallace et al., 2007
E302K	4	Missense	904G > A	Brazilian	Santos et al., 2011
A310G	4	Missense	929C > G	Brazilian	de Lima Santos et al., 2010; Santos et al., 2011
Q312X	4	Nonsense	934C > T	Asian	Nagayoshi et al., 2008
G319fsX341	4	Frame shift	954-955insG	Italian	Lanzara et al., 2004
G320V	4	Missense	959G > T	Multiple	Lanzara et al., 2004; Papanikolaou et al., 2004; Gehrke et al., 2005; Silvestri et al., 2007; Santos et al., 2011
C321W	4	Missense	963C > G	European	Wallace et al., 2007
C321X	4	Nonsense	962G > A, 963C > A	Asian	Huang et al., 2004; Santos et al., 2012
R326X	4	Nonsense	976C > T	Asian	Huang et al., 2004; Papanikolaou et al., 2004
S328fsX337	4	Frame shift	980-983 delTCTC	Slovakian	Gehrke et al., 2005
R335Q	4	Missense	1004G > A		Wallace et al., 2007
C361fsX366	4	Frame shift	1080delC	European	Papanikolaou et al., 2004
N372D	4	Missense	1114A > G		Wallace et al., 2007
R385X	4	Nonsense	1153C > T	Italian	Lanzara et al., 2004; Santos et al., 2012

## BMP-SMAD signaling via HJV is a central regulator of hepcidin

**Figure 1:**
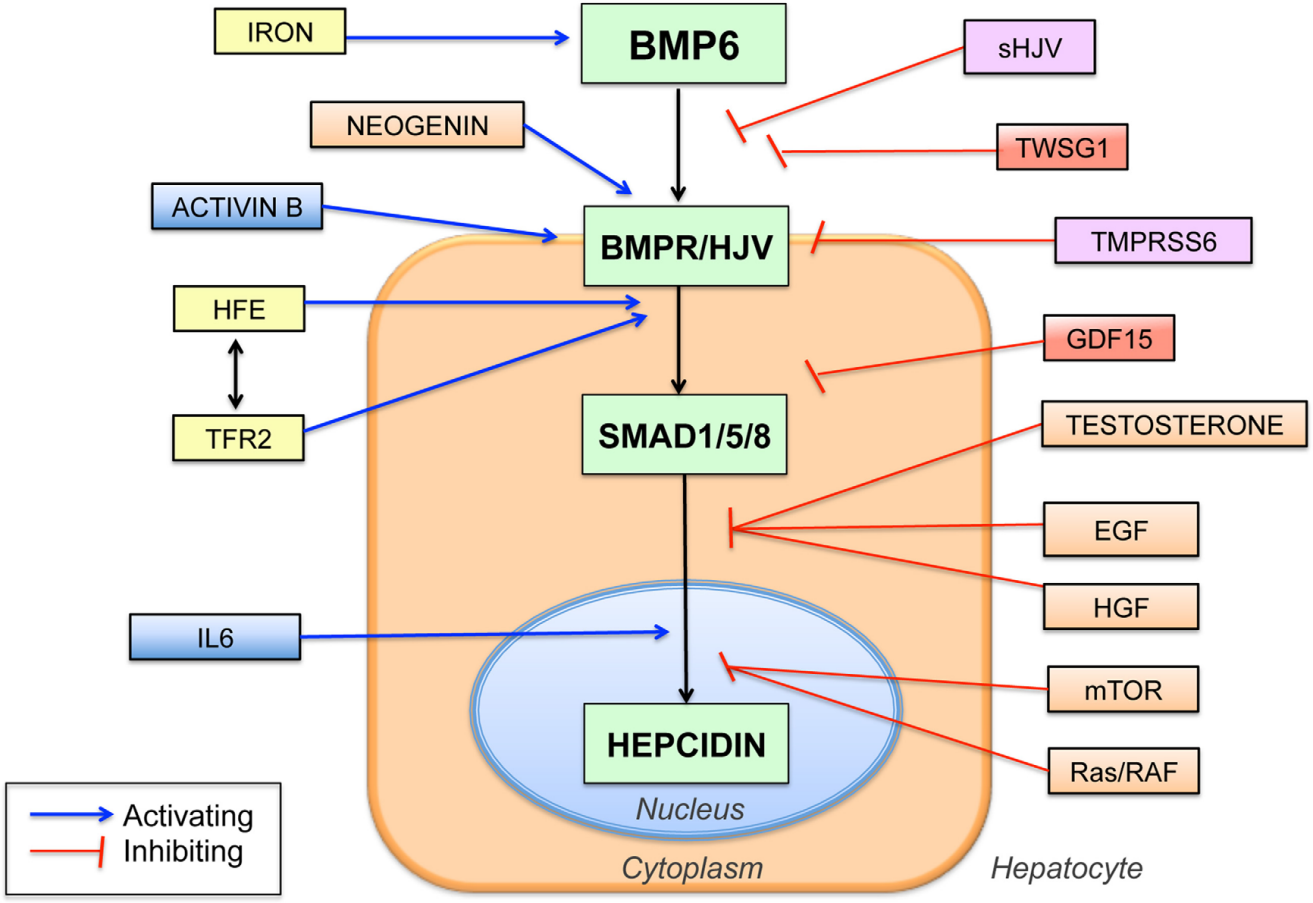
**Schematic diagram showing the central role of the BMP6-HJV-SMAD signaling pathway in hepcidin regulation and the proposed interaction with other hepcidin regulators**. BMP6 binds to the BMP type I and type II receptors (BMPR) and the co-receptor HJV to increase phosphorylation of SMAD1, SMAD5, and SMAD8 proteins (SMAD1/5/8), which translocate to the nucleus to increase hepcidin transcription. Numerous other hepcidin regulators have been identified, many of which are proposed to intersect with the central BMP6/HJV/SMAD pathway at various levels as shown. Proposed iron-mediated hepcidin regulators are shown in yellow, inflammatory mediators in blue, iron deficiency mediators in purple, and anemia mediators in red. Abbreviations: TFR2, transferrin receptor 2; IL6, interleukin 6, sHJV, soluble hemojuvelin, TWSG1, twisted gastrulation 1, GDF15, growth and differentiation factor 15, TMPRSS6, transmembrane serine proteinase 6, EGF, epidermal growth factor, HGF, hepatocyte growth factor, mTOR, mammalian target of rapamycin.

A breakthrough in understanding the mechanism of action of HJV in hepcidin regulation came when HJV was discovered to function as a co-receptor for the bone morphogenetic protein (BMP) signaling pathway (Babitt et al., 2006), analogous to its RGM family homologs (Babitt et al., 2005; Samad et al., 2005). Importantly, this BMP signaling function of HJV was demonstrated to be crucial for its role in regulating hepcidin expression (Babitt et al., 2006) (Figure 1).

BMPs belong to the Transforming Growth Factor-beta (TGF-β) superfamily of ligands (Shi and Massagué, 2003). In the canonical signaling pathway, BMP ligands bind to type I and type II serine threonine kinase receptors to induce phosphorylation of cytoplasmic SMAD1, SMAD5, and SMAD8 proteins. These SMAD proteins form a complex with SMAD4 and translocate to the nucleus to regulate gene transcription. This signaling pathway is further regulated at multiple levels in order to generate a precise signal in a specific cellular context (Shi and Massagué, 2003).

HJV and other RGM family members function as BMP co-receptors that bind selectively to BMP ligands and receptors to enhance SMAD phosphorylation in response to BMP signals (Babitt et al., 2005, 2006; Samad et al., 2005). All RGMs share the ability to bind to the BMP2/BMP4 subfamily and enhance BMP2/BMP4 signaling (Babitt et al., 2005, 2006; Samad et al., 2005; Wu et al., 2012). Moreover, all RGMs utilize BMP type I receptors ALK2, ALK3, and ALK6, and allow preferential signaling through the BMP type II receptor ACTRIIA (Xia et al., 2007, 2008, 2010). However, HJV is unique from other RGMs in that it exhibits preferential ability to bind to the BMP5/BMP6/BMP7 subfamily compared with RGMA and RGMB (Wu et al., 2012).

The BMP-HJV-SMAD signaling pathway activates hepcidin transcription directly through specific BMP-responsive elements (BMP-REs) on the hepcidin promoter (Casanovas et al., 2009; Truksa et al., 2009a). A mutation in the proximal BMP-RE was associated with a more severe iron overload phenotype in a patient with classical *HFE* hemochromatosis, demonstrating its importance in hepcidin regulation in humans (Island et al., 2009). In mice, liver-specific disruption of *Smad4*, the BMP receptors type I *Alk2* or *Alk3*, or the ligand *Bmp6* result in hepcidin deficiency and iron overload, supporting the important role of these specific BMP-SMAD pathway components, in conjunction with HJV, in hepcidin regulation *in vivo* (Wang et al., 2005; Andriopoulos et al., 2009; Meynard et al., 2009; Steinbicker et al., 2011a).

## Soluble HJV

In addition to the GPI-anchored membrane form of HJV, endogenous soluble HJV (sHJV) protein is detectable in human and rodent serum. (Lin et al., 2005; Zhang et al., 2007; Chen et al., 2013). Multiple mechanisms have been proposed for endogenous sHJV generation, including cleavage by the pro-protein convertase furin and the type II transmembrane serine protease TMPRSS6 (Kuninger et al., 2008; Lin et al., 2008; Silvestri et al., 2008a,b). Whereas membrane HJV is a co-receptor for the BMP signaling complex (Babitt et al., 2006), sHJV can antagonize BMP signaling, presumably by binding and sequestering BMP ligands from interacting with cell-surface BMP type I and type II receptors (Babitt et al., 2007) (Figure 1). Indeed, the relative binding affinity of HJV for various BMP ligands roughly correlated with the ability of sHJV to inhibit their biological activity (Babitt et al., 2007; Wu et al., 2012).

Although exogenous sHJV inhibits BMP-SMAD signaling, the source, amount, and physiologic role(s) of endogenously produced sHJV *in vivo* are not well-understood. There is some evidence suggesting that endogenous sHJV is increased by iron deficiency and reduced by iron loading (Lin et al., 2005; Zhang et al., 2007; Silvestri et al., 2008a; Brasse-Lagnel et al., 2010; Chen et al., 2013). Interestingly, the furin cleaved form of sHJV appears to be more potent to inhibit BMP signaling and hepcidin compared with the TMPRSS6-cleaved form (Maxson et al., 2010). Whether HJV cleavage mainly represents a mechanism to remove the activating effects of liver membrane HJV, or whether endogenous sHJV has a direct BMP-SMAD inhibiting effect remains uncertain.

## Extra-hepatic functions of HJV

In addition to the liver, *HJV* mRNA is also highly expressed in skeletal muscle and heart (Niederkofler et al., 2004; Papanikolaou et al., 2004), and has been detected in other tissues (Rodriguez Martinez et al., 2004, Rodriguez et al., 2007; Gnana-Prakasam et al., 2009; Luciani et al., 2011). Tissue specific differences in HJV mRNA regulation and HJV protein glycosylation patterns have also been described (Niederkofler et al., 2005; Fujikura et al., 2011). It was previously hypothesized that skeletal muscle and/or heart could serve as a source of sHJV to suppress hepcidin synthesis in response to iron deficiency or hypoxia (Lin et al., 2005; Zhang et al., 2005). However, mice with a specific knockout of *Hjv* in skeletal ± cardiac muscle do not have altered hepcidin expression or systemic iron balance, at least under basal conditions or with dietary iron changes (Chen et al., 2011; Gkouvatsos et al., 2011). Whether strenuous exercise or hypoxia may uncover a role for muscle hemojuvelin remains uncertain. In contrast, hepatocyte specific *Hjv* knockout mice exhibit an iron overload phenotype similar to global *Hjv* knockout mice (Chen et al., 2011; Gkouvatsos et al., 2011). Thus, hepatic expression of HJV appears to have the most important physiologic role in systemic iron homeostasis regulation *in vivo*.

## Iron stimulates BMP-SMAD signaling to regulate hepcidin

Iron regulates the activity of the BMP6-SMAD pathway to modulate hepcidin expression. Both circulating and liver iron appear to stimulate this pathway through different mechanisms (Ramos et al., 2011; Corradini et al., 2011a). In mice, liver iron content is positively correlated with liver *Bmp6* mRNA levels and overall activity of the Smad signaling pathway (Kautz et al., 2008; Corradini et al., 2011a). Moreover, hepcidin induction by iron is inhibited by a neutralizing BMP6 antibody (Corradini et al., 2011a). These data suggest that liver iron modulates BMP6-SMAD signaling and hepcidin expression at least in part by regulating expression of *BMP6* mRNA (Figure 1). It appears that liver iron regulates BMP6 expression mainly in nonparenchymal cells (Enns et al., 2013), and that iron loading in specific liver cell types may important for this regulation (Daba et al., 2013). However, the mechanism by which hepatic iron levels regulate BMP6 remains unknown. Notably, hepcidin is still increased to a lesser extent by chronic iron loading in *Bmp6* and *Hjv* knockout mice, suggesting that these pathways do not completely account for hepcidin regulation by chronic iron loading (Ramos et al., 2011; Gkouvatsos et al., 2014).

Increases in circulating iron stimulate SMAD1/5/8 phosphorylation and hepcidin expression without affecting *Bmp6* mRNA levels (Corradini et al., 2011a). How circulating iron activates SMAD1/5/8 phosphorylation is unknown, but may involve an interaction with other proteins that are mutated in adult-onset hereditary hemochromatosis (see section HFE and TFR2). HJV liver membrane protein expression itself does not appear to be regulated by iron (Krijt et al., 2012).

Iron administration and BMP6-SMAD signaling also up-regulate inhibitory SMAD7 and SMAD6, and TMPRSS6 (see section TMPRSS6), that can act as feedback inhibitors of BMP-SMAD signaling and hepcidin expression (Kautz et al., 2008; Mleczko-Sanecka et al., 2010; Meynard et al., 2011; Corradini et al., 2011a; Vujić Spasić et al., 2013). It has been hypothesized that these pathways may help prevent excessive hepcidin increases by iron to provide tight homeostatic control (Meynard et al., 2011; Corradini et al., 2011a).

## Interaction of HJV and the BMP-SMAD signaling pathway with other hepcidin regulators

### HFE and TFR2

Adult-onset hereditary hemochromatosis is a less severe iron-overload disorder that manifests later in life compared with JH, and is associated with mutations in *HFE* or *TFR2* (encoding transferrin receptor 2) (Pietrangelo, 2010). Liver expression of HFE and TFR2 are clearly important for iron homeostasis regulation because mice with a hepatocyte-specific knockout of either gene have a similar iron-overload phenotype compared with global *Hfe* or *Tfr2* knockout mice (Wallace et al., 2007; Vujić Spasić et al., 2008). Moreover, liver transplantation corrects much of the *HFE* hemochromatosis phenotype (Garuti et al., 2010; Bardou-Jacquet et al., 2014). Liver hepcidin expression is inappropriately low in mice and humans with *HFE* or *TFR2* mutations, suggesting that both HFE and TFR2 positively regulate liver hepcidin expression (Ahmad et al., 2002; Fleming et al., 2002; Bridle et al., 2003; Muckenthaler et al., 2003; Kawabata et al., 2005; Nemeth et al., 2005; Piperno et al., 2007). HFE and TFR2 are also postulated to function in iron sensing by the liver. The current working model is that when iron-bound transferrin increases in circulation, it binds to transferrin receptor 1 (TFR1) and displaces HFE, which then signals by some mechanism to stimulate hepcidin expression, possibly through an interaction with TFR2 (Schmidt et al., 2008; Gao et al., 2009).

It has been proposed that HFE and TFR2 may form a “supercomplex” with HJV to stimulate hepcidin expression via the BMP-SMAD pathway. Studies supporting this model have demonstrated that liver BMP-SMAD signaling is impaired in mice and humans with *HFE* and/or *TFR2* mutations, suggesting an interaction at some level between HFE, TFR2 and the BMP-SMAD pathway (Corradini et al., 2009, 2011b; Kautz et al., 2009; Wallace et al., 2009; Bolondi et al., 2010; Ryan et al., 2010). Recently, it was published in an overexpression tissue culture system using tagged proteins that HFE and TFR2 can form a complex with HJV (D'Alessio et al., 2012). However, it is not been shown whether these proteins endogenously interact *in vivo*. Moreover, the more severe iron overload phenotype of *HJV* mutations and combined *HFE/TFR2* mutations compared with either *HFE* or *TFR2* mutations alone suggest that the function of these proteins is not entirely overlapping (Pietrangelo et al., 2005; Wallace et al., 2009). Thus, while it appears that HFE and TFR2 interact at some level with the BMP-HJV-SMAD pathway to regulate liver hepcidin expression (Figure 1), the precise molecular mechanisms of how HFE and TFR2 contribute to hepcidin regulation remain an active area of investigation.

### The inflammatory pathway

In addition to iron, inflammatory stimuli also induce hepcidin expression (Ganz, 2013). The most well-characterized pathway is through IL6 activating the Janus kinase JAK2 to phosphorylate STAT3, which then activates the hepcidin promoter directly via a STAT3-binding motif (Wrighting and Andrews, 2006; Pietrangelo et al., 2007; Verga Falzacappa et al., 2007).

Although inflammation downregulates liver *Hjv* mRNA expression (Krijt et al., 2004; Niederkofler et al., 2005; Constante et al., 2007), liver SMAD1/5/8 signaling is often activated in the context of inflammation (Theurl et al., 2011) and is essential for hepcidin regulation by inflammation. Indeed, blocking BMP signaling with a small molecule BMP type I receptor inhibitor or a sHJV recombinant protein inhibits IL6-induced hepcidin expression in cell culture (Babitt et al., 2007; Yu et al., 2008). Moreover, mice with a hepatocyte-specific knockout of *Smad4* exhibit blunted hepcidin response to IL6 treatment (Wang et al., 2005). Importantly, BMP pathway inhibitors lower hepcidin, increase iron availability for erythropoiesis, and ameliorate anemia in animal models of anemia of inflammation (Theurl et al., 2011; Steinbicker et al., 2011b; Sun et al., 2013).

At least two mechanisms are proposed to account for the crosstalk between the BMP-SMAD and IL6-STAT3 pathways in hepcidin regulation. First, there may be an interaction at the level of the hepcidin promoter, where the proximal BMP-RE and the STAT3 binding site are in close proximity (Figure 1). In support of this hypothesis, mutation of the proximal BMP-RE impairs hepcidin promoter activation not only by BMPs, but also by IL6 (Casanovas et al., 2009). Second, inflammation induces hepatic expression of another TGF-β superfamily member, Activin B, which can stimulate hepcidin expression by activating SMAD1/5/8 signaling in hepatoma-derived cell cultures (Besson-Fournier et al., 2012) (Figure 1). Whether Activin B contributes to hepcidin regulation by inflammation *in vivo* remains to be determined.

### TMPRSS6

The serine protease TMPRSS6 has been implicated in hepcidin inhibition by iron deficiency. Mutations in *TMPRSS6* are linked to IRIDA associated with inappropriately high hepcidin levels (Du et al., 2008; Finberg et al., 2008; Folgueras et al., 2008). Moreover, genome-wide association studies have linked common single nucleotide polymorphisms in *TMRPSS6* to iron status and hemoglobin level, supporting an important role for TMPRSS6 in regulating systemic iron homeostasis and normal erythropoiesis (Benyamin et al., 2009; Chambers et al., 2009; Tanaka et al., 2010). TMPRSS6 is proposed to regulate hepcidin expression through an interaction with HJV and the BMP-SMAD pathway in the liver. Specifically, when both proteins are overexpressed in cell culture, TMPRSS6 binds and cleaves HJV to generate sHJV, thereby inhibiting BMP-SMAD signaling (Silvestri et al., 2008b) (Figure 1). In mouse models, the combined deficiency of *Hjv* or *Bmp6* and *Tmprss6* causes iron overload, suggesting that there is a genetic interaction between TMPRSS6 and the BMP6-HJV-SMAD pathway (Truksa et al., 2009b; Finberg et al., 2010; Lenoir et al., 2011). Interestingly, liver membrane expression of Hjv is decreased (Krijt et al., 2011), and serum sHjv levels are unchanged (Chen et al., 2013), in *Tmprss6* knockout mice compared with wildtype mice, which seem contrary to the proposed hypothesis that TMPRSS6 acts to cleave HJV from the liver membrane surface. Future work is needed to fully understand the mechanism of action of TMPRSS6 in hepcidin regulation and iron homeostasis *in vivo.*

### Neogenin

In addition to TMPRSS6, the deleted in colorectal cancer (DCC) family member neogenin is also proposed to function as an HJV interacting protein that modifies BMP-SMAD signaling and iron homeostasis (Figure 1). In particular, neogenin binds to HJV, like other RGM family members (Matsunaga et al., 2004; Zhang et al., 2005; Conrad et al., 2010). Moreover, neogenin mutant mice exhibit reduced hepcidin levels and iron overload consistent with a role for neogenin in regulating hepcidin and systemic iron balance *in vivo* (Lee et al., 2010). However, the mechanism of action of neogenin in hepcidin and iron homeostasis regulation is still not fully understood. In some studies, neogenin increased HJV cleavage (Enns et al., 2012), while in other studies, neogenin reduced HJV secretion (Lee et al., 2010). Moreover, neogenin was variably shown to inhibit (Hagihara et al., 2011), have no effect (Xia et al., 2008), or stimulate BMP signaling (Lee et al., 2010). Whether neogenin and HJV interact in a cell autonomous or cell non-autonomous manner *in vivo* remains unclear, and how this interaction occurs may be important for downstream functional effects.

### Other pathways

Hepcidin suppression by erythropoietic drive appears to be mediated by secreted factor(s) released by proliferating red blood cell precursors in the bone marrow (Pak et al., 2006; Vokurka et al., 2006). Two proposed erythroid hepcidin regulators are the TGF-β /BMP superfamily modulators growth and differentiation factor 15 (GDF15) and twisted gastrulation 1 (TWSG1), at least in the context of ineffective erythropoiesis in iron loading anemias (Tanno et al., 2007, 2009) (Figure 1). The role of GDF15 and TWSG1 in hepcidin suppression by erythropoietic drive in other contexts has been questioned (Ashby et al., 2010; Casanovas et al., 2013). Recently, erythroferrone has been proposed as a novel erythroid regulator (Kautz et al., 2013), but its mechanism of action is not yet reported.

A number of other hormones, growth factors and signaling pathways have recently been implicated in hepcidin regulation including testosterone, estrogen, hepatocyte growth factor (HGF), epidermal growth factor (EGF), endoplasmic reticulum stress, gluconeogenic signals and the Ras/RAF and mTOR signaling pathways (Oliveira et al., 2009; Vecchi et al., 2009, 2014; Goodnough et al., 2012; Hou et al., 2012; Yang et al., 2012; Guo et al., 2013; Latour et al., 2014; Mleczko-Sanecka et al., 2014). Notably, the majority of these pathways appear to regulate hepcidin through an intersection with the BMP-SMAD pathway at some level (Goodnough et al., 2012; Guo et al., 2013; Latour et al., 2014; Mleczko-Sanecka et al., 2014) (Figure 1).

## Conclusion

Understanding the genetic basis for JH has yielded important insights into the molecular mechanisms of systemic iron homeostasis. Hepcidin and its receptor ferroportin are key regulators of body iron balance, and the BMP-SMAD pathway via the co-receptor HJV is a central regulator of hepcidin production (Figure 1). Knowledge of these pathways has already lead to the development of novel therapeutic strategies that target the molecular mechanisms underlying iron homeostasis disorders, with several new treatments currently being evaluated in human clinical trials (Fung and Nemeth, 2013). Future work will be needed to fully understand the mechanisms by which iron levels are sensed by the liver and integrated with other pathways to regulate BMP-SMAD signaling, hepcidin expression, and systemic iron homeostasis.

### Conflict of interest statement

Jodie L. Babitt has ownership interest in a start-up company FerruMax Pharmaceuticals, which has licensed technology from the Massachusetts General Hospital based on the work cited here and in prior publications. All other authors declare the absence of any commercial or financial relationships that could be construed as a potential conflict of interest.
